# Recent Advances in the Research on Luotonins A, B, and E

**DOI:** 10.3390/molecules29153522

**Published:** 2024-07-26

**Authors:** Ján Gettler, Martin Markovič, Peter Koóš, Tibor Gracza

**Affiliations:** Department of Organic Chemistry, Institute of Organic Chemistry, Catalysis and Petrochemistry, Slovak University of Technology, Radlinského 9, SK-812 37 Bratislava, Slovakia; jan.gettler@stuba.sk (J.G.); tibor.gracza@stuba.sk (T.G.)

**Keywords:** luotonins A, B, E, quinolinopyrroloquinazoline, alkaloids, cytotoxicity, biological activity

## Abstract

This digest review summarises the most recent progress in the study on luotonins A, B and E. The literature covered in this overview spans from January 2012 to April 2024 and presents synthetic methodologies for the assembly of the quinolinopyrrolo-quinazoline scaffold, the structural motifs present in luotonins A, B, and E, and the evaluation of the biological activities of their derivatives and structural analogues.

## 1. Introduction

**Figure 1:**
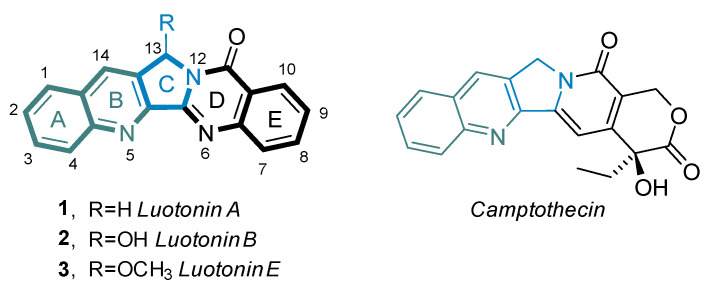
Structures of luotonins A, B, and E and camptothecin.

Since the discovery of luotonins in the *Peganum nigellastrum* Bunge (*Zygophyllaceae*), there has been ongoing interest in the synthesis and biological evaluation of these intriguing compounds [1,2,3]. Luotonins were isolated from the aerial parts of plants that have been long used in Chinese traditional medicine for treating rheumatism, abscesses, and diseases accompanied by inflammation. All isolated luotonin alkaloids (luotonins A-F) have shown moderate cytotoxic activity against selected cancer cell lines [4,5,6,7,8]. Among them, luotonin A (**1**) displays promising cytotoxicity against murine leukaemia P-388 cells (IC_50_ 1.8 mg/mL) facilitated by stabilising the topoisomerase I and DNA complex [9], similar to the structurally related topo inhibitor, camptothecin [10] (Figure 1). Although this alkaloid is not sufficiently active for cancer chemotherapy, it has become an interesting lead structure. In this context, there is still ongoing interest in the construction of the quinolinopyrrolo-quinazoline scaffolds and related frameworks. Numerous successful synthetic approaches to this class of compounds have appeared over the last decades. In 2011, Jahng and coworkers [11] published an account of the biological activities and syntheses of luotonins A-F. The presented review covers the latest findings on the synthesis of luotonins A, B, and E as well as their derivatives and structural analogues and the evaluation of their biological activities over the period from January 2012 to April 2024. 

## 2. Synthesis and Biological Activity

The publications discussed in this review are divided according to the synthetic strategies used in constructing the pentacyclic structural core of the luotonins.

### 2.1. Synthesis of Luotonin A via Construction of the 5H-pyrrolo[4,3-b]pyridine Core (B and C Rings)

The most frequently applied synthetic strategy for the construction of the quinolinopyrrolo-quinazoline skeleton during the reviewed period uses *aza*-Diels–Alder addition as a key step in the synthesis of target compounds. 

**Scheme 1:**
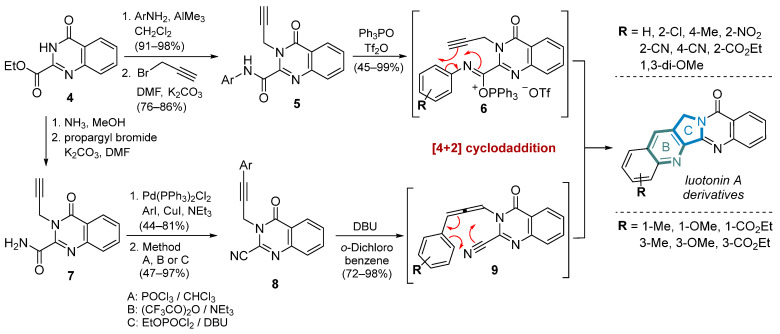
Haider’s syntheses of luotonin A and its derivatives.

Haider and coworkers [12,13,14] reported two synthetic pathways starting from ethyl 4-oxo-3,4-dihydroquinazoline-2-carboxylate **4**, which allow the selective introduction of various substituents into all available positions of ring A of luotonin A. The first paper [12] describes the synthesis of luotonin A and its substituted derivatives by improving Yao’s synthetic route starting from anthranilamide [15]. The anilide-type key intermediates **5** were prepared from ethyl 4-oxo-3,4-dihydroquinazoline-2-carboxylate **4** in two steps by applying Weinreb’s amidation method, providing products in good yields (Scheme 1). Subsequently, the [4+2] cycloaddition reaction of requisite intermediate **6**, generated in situ from **5** by treatment with Hendrickson’s reagent provided the pyrrolo[4,3-*b*]pyridine core (B and C rings of **1**). Alternatively, the simultaneous assembly of B and C rings of the luotonin A skeleton was achieved from carbonitriles **8** [13] by employing Wang’s conditions (heating in 1,2-dichlorobenzene at 110–120 °C in the presence of 5 mol % DBU) [16]. This three-step sequence, DBU-mediated alkyne/allene isomerisation, intramolecular [4+2] cycloaddition reaction of allene **9**, and a 1,5-hydrogen shift in the primary cycloadduct then produces the aromatic quinoline scaffold. Arylpropargyl-substituted quinazolinonecarbonitriles **8** were obtained by the Sonogashira coupling of appropriate aryl iodides with 4-oxo-3-propargyl-3,4-dihydroquinazoline-2-carboxamides **7** and the subsequent dehydration reaction of the amides. This method is suitable for the selective introduction of both electron-donating as well as electron-withdrawing substituents into position 1 or position 3 of the alkaloid compared to a previously used cycloaddition approach that gave access to 2- or 4-substituted luotonin A derivatives (Scheme 1).

Preliminary in vitro screening (using the Resazurin assay [17]) for antiproliferative activity showed that thiophene isostere of luotonin A is practically inactive, whereas the 1,3-dimethoxy derivative exhibits a slightly higher activity profile than the lead compound, luotonin A. The EC_50_ value of 1,3-dimethoxy-luotonin A towards the Ishikawa cell line (ECACC 99040201, endometrial carcinoma) is 5.84 µM (luotonin A: no inhibition), while the EC_50_ value of 1,3-dimethoxy derivative towards the MDA-MB-231 cell line (ATCC HTB-26, triple-negative breast cancer) is 31.82 µM (luotonin A: >100 µM). The EC_50_ value of 1,3-dimethoxy derivate towards the other cell lines (KB/HeLa, NCI-H460, A2780, MDA-MB-435S) was >100 µM.

**Figure 2:**
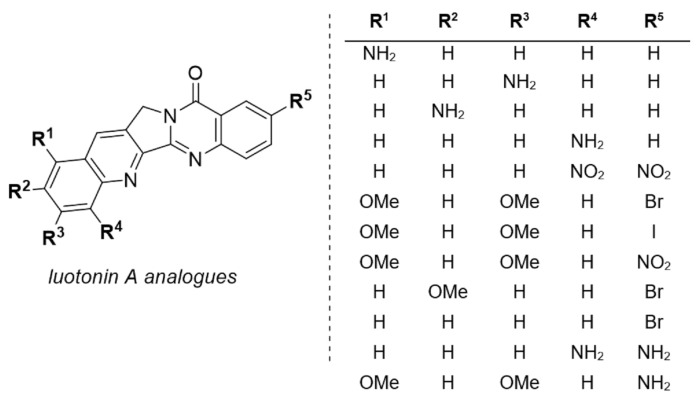
Structures of A and E substituted analogues of luotonin A.

Later, Haider et al. [18,19] prepared a series of luotonin A analogues with modification to the A and E rings using the Povarov reaction as a key step (Figure 2). The evaluation of the antiproliferative activity towards human tumour cell lines revealed the 4-amino and 4,9-diamino compounds to be the most effective agents, showing an interesting profile of cytotoxic activity. Specifically, 4,9-diamino-luotonin A (IC_50_ of 2.03 µM [95% CI = 1.79–2.29] against SW480 cells and an IC_50_ of 0.82 µM [95% CI = 0.71–0.94] against HL60 cells) is a potent antiproliferative agent that is active in a similar concentration range as clinically used topo I inhibitors.

**Scheme 2:**
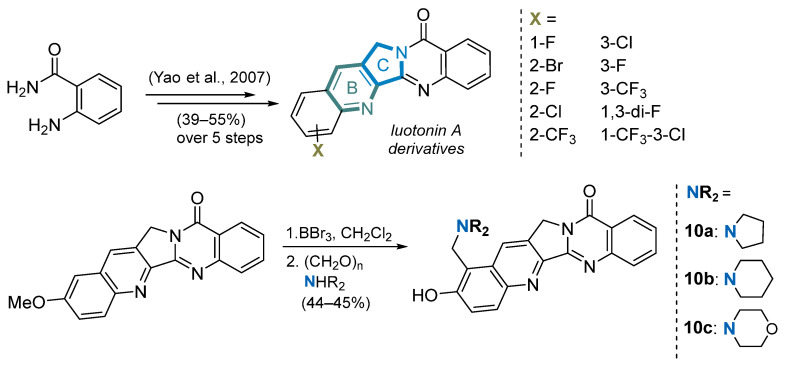
Synthesis of luotonin A derivatives by Liu and Wang.

Luotonin A and its derivatives have also attracted attention as potential fungicides, insecticides [20], and pesticides [21]. Liu and coworkers [20] reported on the fungicidal and insecticidal activity of halo-substituted derivatives of **1**. The target compounds were synthesised from anthranilamide using Yao’s synthetic strategy [15], applying an intramolecular *aza*-Diels–Alder reaction for the formation of the 5*H*-pyrrolo[4,3-*b*]pyridine core (B and C rings) (Scheme 2). To obtain more diversified modifications at the C-13 position of the pentacyclic skeleton, 1-methoxy luotonin A was treated with boron tribromide and subsequently subjected to a Mannich reaction with different amines to produce compounds **10a**–**10c**.

Novel luotonin A derivatives were tested for their activity against *Botrytis cinerea*, *Magnaporthe oryzae*, and *Aphis craccivora*. The results demonstrated that most of the luotonin A analogues displayed excellent in vitro biological activity against *B. cinerea*. Compared with the luotonin A (0.25 mM), 2-methoxy (0.18 mM), 2,3-dimethoxy (0.19 mM), 2,3-difluoro (0.17 mM), **10b** (0.16 mM) and **10c** (0.09 mM) compounds displayed the greatest antifungal activity. In particular, the in vitro antifungal activity of 1-morpholinomethyl derivative **10c** is comparable to the positive control (azoxystrobin, 0.09 mM). The 3-fluoro and 3-trifluoromethyl analogues of **1** were the most active compounds against *A. craccivora*, each showing the same mortality values of 42.05% at 50 mg/mL. 

Wang and coworkers [21] evaluated some analogues of **1** for their antiviral activities and fungicidal activities. Natural luotonin A **1** displayed similar antiviral activity (inhibitory rates of 41%, 45%, and 36% at 500 μg/mL for inactivation, curative, and protection activities in vivo, respectively) to ribavirin (inhibitory rates of 40%, 37%, and 40% at 500 μg/mL for inactivation, curative, and protection activities in vivo, respectively). However, the effect of introducing both EWG and/or electron-donating groups to the A-ring on the activity is not obvious. The 2,3-dimethoxy and 2-chloro derivatives exhibited about similar levels of antiviral activities, while the activity of 2-methyl compounds was slightly lower.

**Scheme 3:**
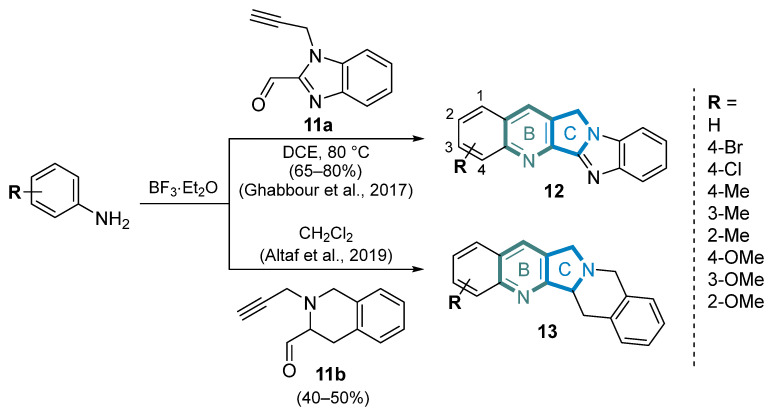
Synthesis of de-carbonyl luotonin derivatives **12** and tetrahydrobenzo[6,7]indolizino[1,2-*b*]quinolines **13** via an intramolecular Povarov reaction.

Menéndez, Arumugam, Kumar, and coworkers [22,23] described the design, synthesis and biological study of a new class of luotonin A analogues **12** and **13**, which differ from the parent compound by lacking the D-ring carbonyl substituent (Scheme 3). The structures of benzimidazole-fused **12** and tetrahydroquinoline-fused pyrrolo[3,4-*b*]quinolines **13** were designed based on the predicted mechanism of action of camptothecin on topoisomerase I DNA using the docking of decarbonylated luotonin A onto the topo I. The target compounds **12** and **13** were synthesised from readily available 1-propargyl-benzimidazole-2-carbaldehyde **11a** [22] and 1-propargyl-tetrahydroquinoline **11b** [23]. Thus, boron trifluoride diethyl etherate catalysed intramolecular Povarov reactions using various substituted arylamines provided desired products in good to excellent yields.

Prepared de-carbonyl analogues of luotonin A **12** were evaluated for their topoisomerase I inhibition, as well as their in vitro cytotoxic activity against four highly aggressive human cancer cell lines of different origins (KB, a subline of the HeLa cell line, MDA-MB231, LNCap, and HT1080). The 2-methoxy and 4-methoxy derivatives exhibited a high topo I inhibition activity that is comparable to that of the standard drug camptothecin. In the cytotoxicity assay towards several highly aggressive tumours, it was observed that some of the newly prepared luotonin A analogues showed promising activity. Specifically, the 4-Cl-analogue showed improved cytotoxicity towards the KB cell line compared to the parent alkaloid luotonin A. 

**Scheme 4:**
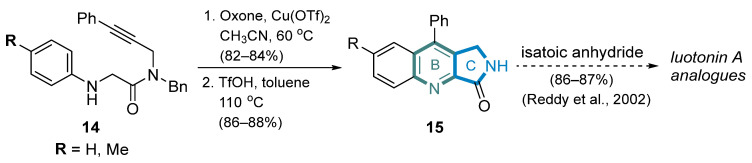
Formal synthesis of 13-aryl-substituted luotonin A.

In 2019, Muthukrishnan and coworkers [24] reported on the novel formal synthesis of 13-aryl-substituted luotonin A derivatives. The key intermediates of Yadav and Reddy synthesis [25], quinoline fused lactams **15**, were obtained from alkyne tethered *N*-benzyl glycine derivatives **14** by oxone-promoted intramolecular dehydrogenative Povarov cyclisation and the subsequent deprotection of an amide under acidic conditions (Scheme 4).

**Scheme 5:**
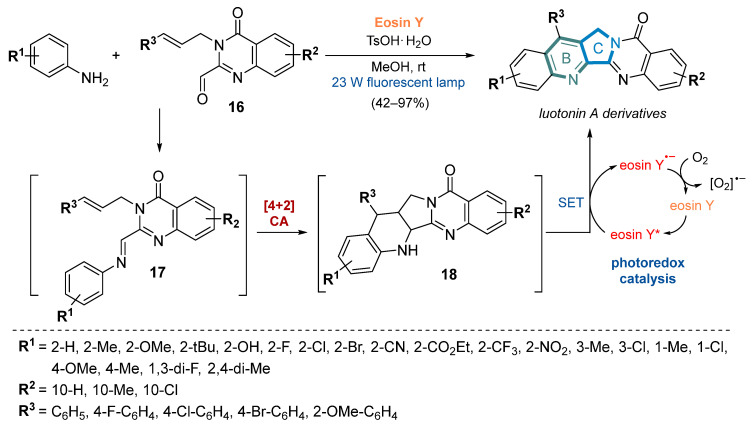
Synthesis of luotonin A derivatives via synergistic visible-light photoredox and acid catalysis.

Recently, a rapid synthesis of luotonin A derivatives using eosin Y photocatalyst and TsOH as a co-catalyst via the Povarov cycloaddition reaction was described [26]. The authors proposed a tentative mechanism for this visible-light-induced transformation (Scheme 5). The intermediate **18** is formed via an intramolecular [4+2] cyclisation of the in situ-generated imine **17** prepared from various anilines and carbaldehydes **16**. The resulting cycloadduct **18** is then oxidised using a system consisting of oxygen and an organic dye eosin Y via SET mechanism, forming the quinoline skeleton of the desired luotonin A derivatives.

**Scheme 6:**
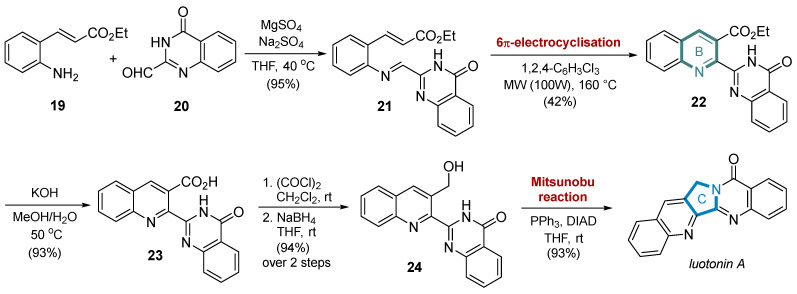
Cheon’s synthesis of luotonin A.

In 2016, Cheon and coworkers [27] developed a new synthesis of luotonin A starting from ethyl-2-aminocinnamate **19** and quinazolinone-2-carbaldehyde **20** in a 33% total yield over six consecutive steps (Scheme 6). The synthetic strategy is based on two key reactions for the successive production of the B and C rings of target luotonin A. The quinoline scaffold containing the B ring of **1** was formed by 6π electrocyclisation of aldimine **21** in 1,2,4-trichlorobenzene under microwave irradiation. Another key intermediate—alcohol **24**—was obtained in three steps involving the basic hydrolysis of ester **22** to carboxylic acid **23**, followed by chlorination with oxalyl chloride and reduction using NaBH_4_. Finally, the total synthesis of luotonin A was completed by the formation of a pyrrole C ring using an intramolecular Mitsunobu reaction of alcohol **24**.

**Scheme 7:**
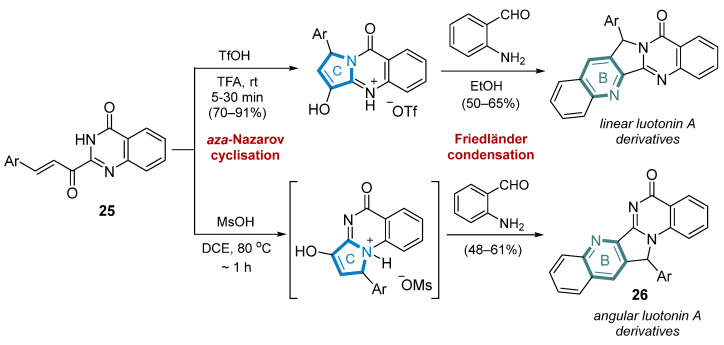
Rassapalli’s synthesis of luotonins A and their regiomers **26**.

Another approach to 12-aryl-substituted luotonins A and their regioisomeric analogues **26** via an acid-mediated *aza*-Nazarov–Friedländer condensation sequence using quinazolinonyl enones **25** was developed by Rassapali and coworkers [28,29] (Scheme 7). The regioselectivity of the Nazarov cyclisation was successfully controlled by the acidic reaction conditions. The test for cytotoxicity and topo I inhibition showed only weak activities compared to adriamycin and camptothecin, respectively.

**Scheme 8:**
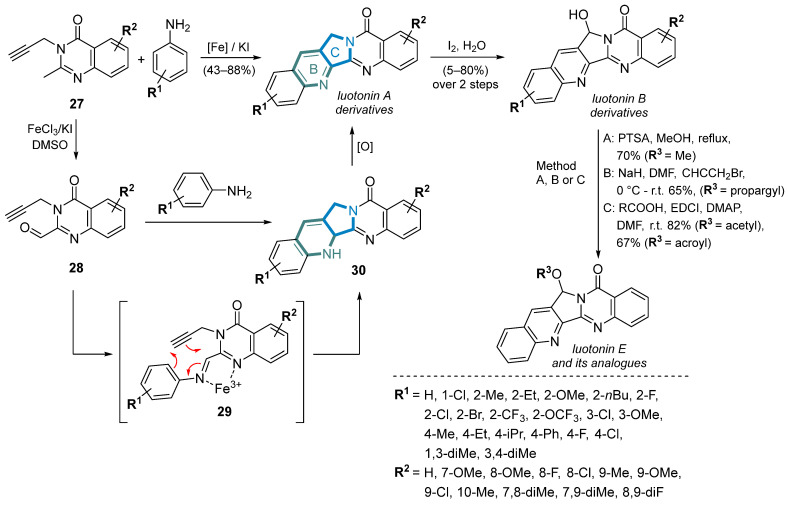
Synthesis of luotonin A, B, and E derivatives.

Very recently, Wu, Zhu, and coworkers [30] developed a versatile synthetic strategy for the synthesis of luotonins A, B, and E and their analogues using synergistic FeCl_3_/KI-catalysed oxidative cyclisation (Scheme 8). The proposed mechanism of this transformation involves the construction of the 5*H*-pyrrolo[4,3-*b*]pyridine core (B and C rings) through an intramolecular [4+2] cycloaddition reaction of imine **29**. This imine is formed in situ from 3-*N*-propargyl-2-methyl-quinazolinones **27** via iodination/Kornblum oxidation in the presence of FeCl_3_/KI, providing aldehyde **28**, which subsequently undergoes condensation with anilines. The FeCl_3_-catalysed cycloaddition then yields pentacyclic intermediate **30** which is aromatised to produce the luotonin A skeleton. Moreover, luotonin A derivatives can also be oxidised using iodine, DMSO, and H_2_O to afford corresponding luotonin B compounds. Further transformation of luotonin B to luotonin E analogues underlines the potential application of this method.

### 2.2. Synthesis of Luotonins A via Construction of the Pyridine Core (B Ring)

**Scheme 9:**
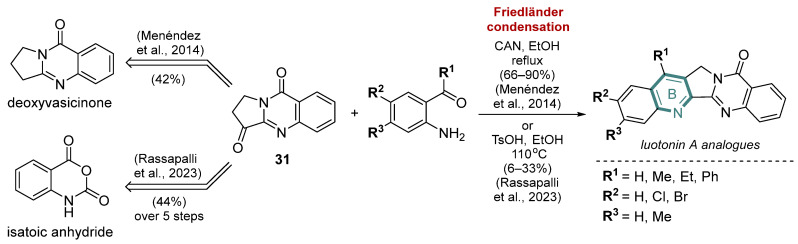
Synthesis of luotonin A analogues via Friedländer reaction.

Friedländer’s synthesis of quinolines has been applied in the synthetic strategies by Menéndez [31] and Rassapalli [32]. The condensation between *o*-amino-benzaldehydes/ketones and corresponding cyclic ketone **31** under acidic conditions (cerium(IV) ammonium nitrate [31] or *p*-toulenesulfonic acid [32]) provided luotonin A derivatives (Scheme 9). The key lactone **31** was obtained from deoxyvasicinone [31] and isatoic anhydride [32], respectively. The A ring-modified analogues, 2-chloro-14-phenyl and 14-(3,5-dimethylphenyl)-substituted luotonin A, showed higher topo I inhibition activity compared to the reference compound **1**. Additionally, the 3-methyl-14-(3,5-dimethylphenyl) derivative of **1** exhibited activity comparable to camptothecin. Furthermore, almost all compounds showed better activity than luotonin A in cell cytotoxicity assays [31]. Docking studies of luotonin A–topoisomerase I DNA ternary complexes revealed that most compounds bound in a similar manner to standard topoisomerase poisons such as topotecan and luotonin A. In most cases, the presence of the substituent on the B ring at the C-14 position induced a better fit to the binding site.

**Scheme 10:**
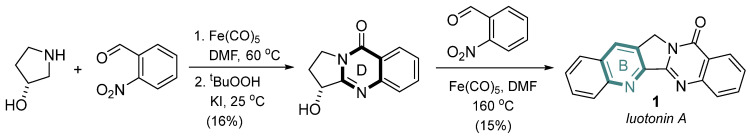
A short synthesis of luotonin A.

In 2020, Chusov et al. [33] described the reductive condensation of *o*-nitrobenzaldehydes with amines utilising iron pentacarbonyl as a reducing agent. Subsequent oxidation of the intermediate led to a great variety of polycyclic nitrogen-containing heterocycles under mild conditions (Scheme 10). This transformation sequence was also employed in a concise synthesis of luotonin A starting from readily available materials such as 3-pyrrolidinole and 2-nitrobenzaldehyde in three operational steps without chromatography. However, the natural alkaloid was isolated in a low overall yield (2% with a 15% yield for the final step).

### 2.3. Synthesis of Luotonins A, B, and E via Construction of the Pyrrole Core (C Ring)

During the reviewed period, several synthetic strategies of luotonins A, B, and E were presented, involving the formation of the pyrrole ring by cyclisation reactions. 

**Scheme 11:**
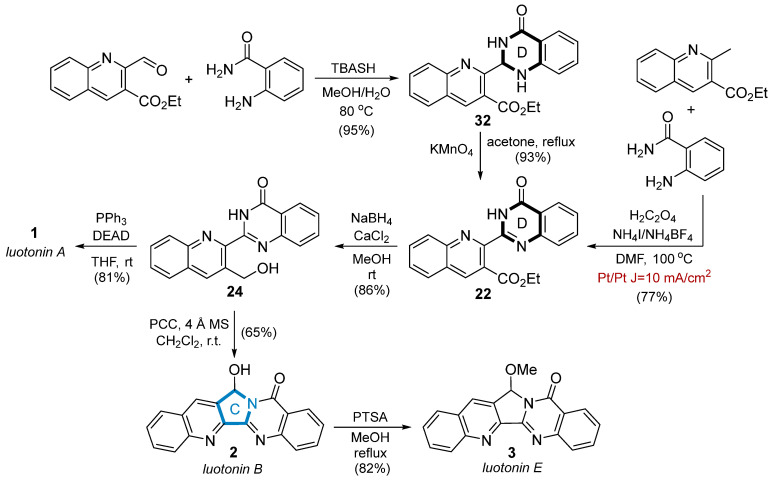
Nagarapu’s and Wang-Zha’s syntheses of luotonin A, B, and E.

Nagarapu and coworkers [34] demonstrated a novel approach for the synthesis of 2-dihydroquinazolin-2-ylquinoline **32** using tetrabutylammonium hydrogen sulphate (TBAHS) as an acidic phase-transfer catalyst. Following oxidation of prepared intermediate **32** using KMnO_4_ provided 2-(4-oxo-3,4-dihydro-quinazolin-2-yl)-quinoline-3-carboxylic acid ethyl ester **22**, which was reduced with NaBH_4_, yielding alcohol **24** (Scheme 11). The pentacyclic skeleton of **1** was accomplished via Mitsunobu cyclisation of alcohol **24**. Alternatively, quinoline derivative **22** was also prepared by iodine-mediated electrochemical C(sp^3^)–H cyclisation [35]. Luotonin B **2** was prepared by the PCC oxidation of intermediate **24** in CH_2_Cl_2_ at a 65% yield. The subsequent treatment of compound **2** with PTSA in methanol provided luotonin E **3** at an 82% yield. Overall, the syntheses of luotonin A, B, and E were accomplished in a direct fashion with high overall yields (57%, 45%, and 37%, respectively).

**Scheme 12:**
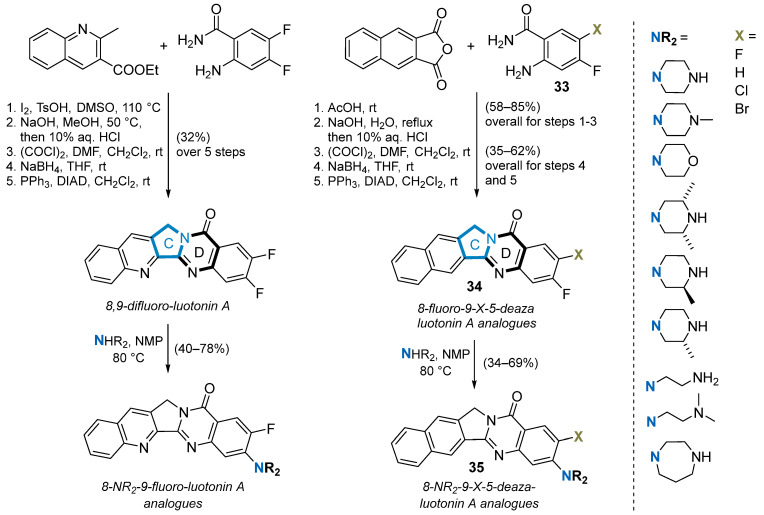
Synthesis of 8-dialkylamino-9-fluoro-luotonin A and 8-dialkylamino-9-halo-5-deaza analogues of luotonin A.

In 2021, Luo et al. [36] published the structure–activity relationship study of 8,9-disubstituted luotonin A analogues and their antiproliferative activity against four cancer cell lines. A series of 8-dialkylamino-9-fluoro luotonin A analogues were prepared in six steps, as outlined in Scheme 12. The quinazolinone intermediate was prepared via an I_2_-catalysed oxidative reaction from 2-amino-4,5-difluorobenzamide and ethyl 2-methylquinoline-3-carboxylate in the presence of TsOH in DMSO. Following the reaction sequence, the basic hydrolysis of the ester group, chlorination using oxalyl chloride, reduction with sodium borohydride in THF, and cyclisation under Mitsunobu reaction conditions yielded 8,9-difluoro-luotonin A in 32% yield over five steps [27]. Subsequent regioselective nucleophilic substitution with various amines provided the targeted 8-dialkylamino-9-fluoro luotonin A analogues in 40–78% yields. 5-Deaza luotonin A analogues **34** and **35** were prepared using the same synthetic route from 2-aminobenzamides **33** and 2,3-naphthalenedicarboxylic anhydride. 

The SAR studies revealed that the in vitro anticancer activity of luotonin A was significantly improved by the introduction of 8-piperazine group [8-piperazinyl-9-fluoro-derivate of **1**: HepG2 (IC_50_ = 3.58 µM), A549 (IC_50_ = 4.85 µM), MCF-7 (IC_50_ = 5.33 µM), and HeLa (IC_50_ = 6.19 µM) cell lines] and the 5-deaza modification [HepG2 (IC_50_ = 1.20 µM), A549 (IC_50_ = 2.09 µM), MCF-7 (IC_50_ = 1.56 µM), and HeLa (IC_50_ = 1.92 µM) cell lines]. Both compounds also exhibited potent topoisomerase I inhibition activity [36]. 

**Scheme 13:**
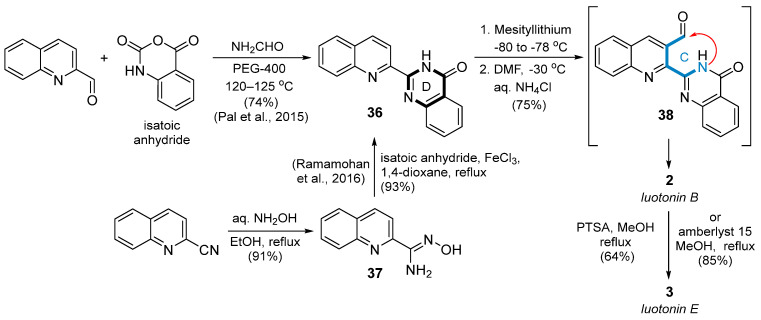
Synthesis of luotonin B and E.

Pal and coworkers [37] developed a general synthesis of the quinazolin-4(3*H*)-one-based alkaloids, including luotonins B and E. The key intermediate, 2-quinolin-2-yl-3*H*-quinazolin-4-one **36**, containing the A, B, D, and E rings of the target compounds, was obtained via a catalyst-free three-component reaction using isatoic anhydride, quinoline-2-carbaldehyde, and formamide (Scheme 13). Following the ortho-lithiation of **36** with mesityllithium, the treatment of in situ-formed lithiated species with DMF afforded luotonin B **2**. The described reaction sequence proceeded via intermediary formed aldehyde **38**, which directly cyclised, forming a pyrrole ring of luotonin B. Luotonin E was prepared by the methylation of **2** in the presence of PTSA. 

Later, Ramamohan et al. [38] described a similar synthetic procedure to luotonins **2** and **3** by applying an iron(III) chloride-catalysed reaction in the synthesis of quinazolin-4(3*H*)-ones. Thus, the key intermediate **36** was prepared from quinoline-2-carbonitrile in two steps (Scheme 13). In the first step, the transformation of the cyano-group of the starting material was achieved using aqueous hydroxylamine in ethanol, providing amidoxime **37** at a 91% yield. Following the reaction of a mixture of carboxamidine **37** and isatoic anhydride, using 10 mol % FeCl_3_ in 1,4-dioxane furnished the desired product **36** at a 93% yield.

**Scheme 14:**
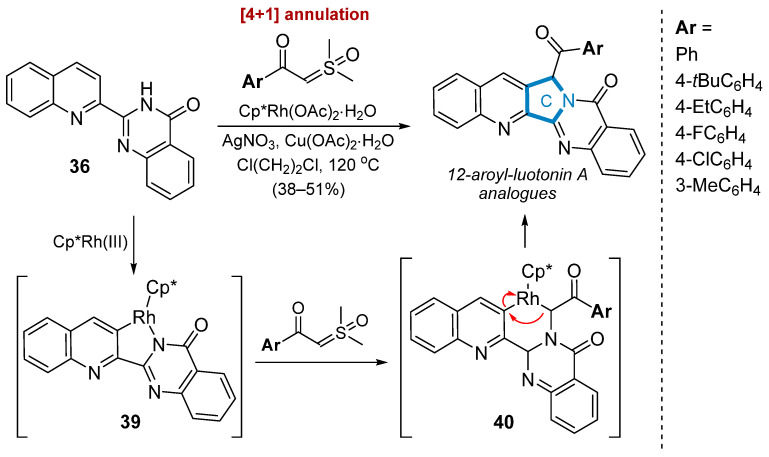
The Rh-catalysed [4+1] annulation in the synthesis of 12-aroyl analogues of luotonin A.

In 2020, Cheng and coworkers [39] described the synthesis of 12-aroyl-substituted derivatives of luotonin A. Their synthetic strategy was based on rhodium catalysed [4+1] annulation between quinazolin-4(3*H*)-ones and sulfoxonium ylides, leading to corresponding quinolino[2′,3′:3,4]pyrrolo[2,1-*b*]quinazolin-11(13H)-ones. Specifically, the 2-quinolin-2-yl-3*H*-quinazolin-4-one **36** undergoes intramolecular cyclisation in the presence of Cp*Rh(OAc)_2_∙H_2_O, AgNO_3_, Cu(OAc)_2_∙H_2_O and aroyl sulfoxonium ylides in dichloroethane, affording 12-aroyl-substituted derivatives of luotonin A with the direct construction of a pyrrole ring (C ring), albeit only in moderate to good yields (Scheme 14).

The proposed reaction mechanism involves the formation of the five-membered rhodacyclic intermediate **39** via C-H activation. Following the insertion of aroyl sulfoxonium ylide to the five-membered intermediate **39** forms a six-membered rhodacycle **40**, which is then transformed via the reductive elimination to furnish the [4+1] annulation product. The formed Rh(I) metal catalyst is oxidised by Cu(II) and Ag(I) to Rh(III) and re-enters the catalytic cycle.

**Scheme 15:**
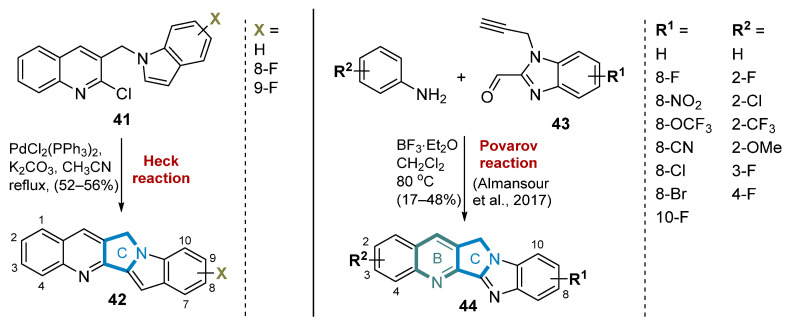
Synthesis of luotonin A analogues.

Zhang, Liu, and coworkers [40] described the syntheses of new luotonin A analogues **42** and **44** from anilines using an intramolecular Heck reaction of 2-chloro-quinolines **41** and the Povarov cyclisation of imine intermediates formed in situ from anilines and aldehydes **43**, respectively (Scheme 15). Prepared compounds were evaluated for their antifungal activities against five plant fungi species. Most of these luotonin A analogues exhibited significant fungicidal in vitro activity against *Botrytis cinerea* with EC_50_ values less than 1 μg/mL. Among them, fluoro-substituted derivatives **44** showed superior antifungal activity against *B. cinerea* (EC_50_ for 4-F-**44**: 0.036 μg/mL, 8-F-**44**: 0.050 μg/mL, 2,8-di-F-**44**: 0.042 μg/mL, 10-F-39: 0.048 μg/mL), which was more potent than boscalid (EC_50_ = 1.790 μg/mL).

**Scheme 16:**
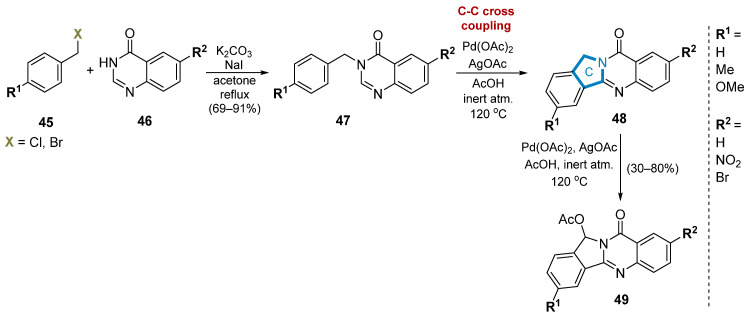
Synthesis of luotonin A analogues [33].

Mondal et al. [41] developed a one-pot protocol to synthesise quinazolinone-fused *N*-heterocyclic compounds **48**, structurally similar to the natural luotonins. The pyrrole ring (C-ring) is formed in a key Pd(OAc)_2_/AgOAc-promoted intramolecular dehydrogenative cross-coupling reaction of 3-aryl quinazolin-4(3*H*)-ones **47**. These starting quinazolines **47** are readily available in good yields via the benzylation of the N-3 centre of quinazolinone **46** using aryl halides **45** in the presence of K_2_CO_3_ in acetone (Scheme 16). In the case of substrate **47**, which bears R^2^ = NO_2_ and Br substituents, the cyclisation product **48** was isolated along with the *O*-Ac product **49** formed by subsequent CH_2_-*N*-oxidation. Compound **49** was shown to exhibit the best results in the study of a concentration-dependent cell viability assay on SiHa cells, a human cervical cancer cell line, with an observed IC_50_ value of 23 μM.

### 2.4. Synthesis of Luotonin A via Construction of the Pyrrolo[1,2-a]pyrimidin-4(6H)-one Core (C a D Rings)

**Scheme 17:**
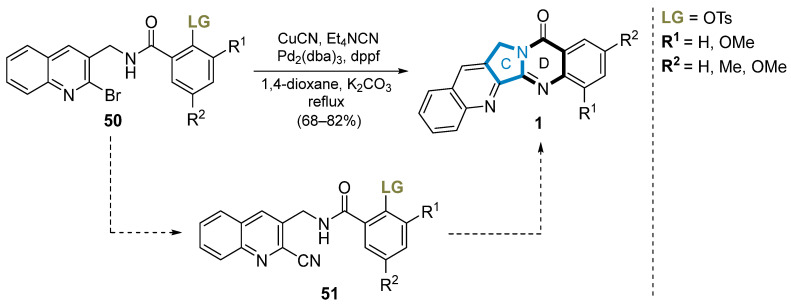
Synthesis of luotonin A and its analogues via intramolecular Diels–Alder reaction.

In 2018, Toyota and coworkers [42] presented an improved synthesis of luotonin A and its analogues based on previous results [43,44]. Similarly, the construction of the pyrrolo[1,2-*a*]pyrimidinone skeleton (C and D rings) was achieved by anion-assisted double intramolecular hetero Diels–Alder transformation. The reaction of amides **50** using Pd_2_(dba)_3_ as a catalyst and DPPF as a ligand together with a mixture of CuCN and Et_4_NCN as a cyanide donor at 130 °C in dioxane provided the desired pentacyclic compounds in good yields (Scheme 17).

**Scheme 18:**
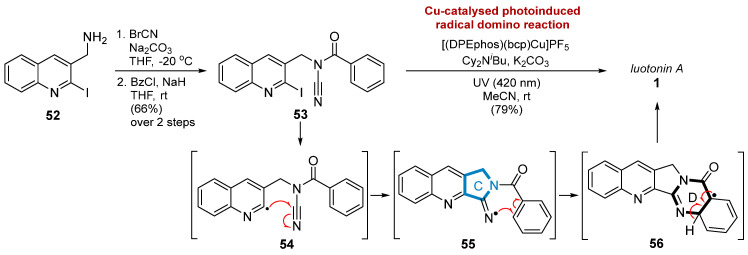
Evano’s synthesis of luotonin A via photoinduced Cu-catalysed cyclisation.

Evano and coworkers [45] described a concise synthesis of luotonin A, in which the pyrrolopyrimidinone fragment (C and D rings) of the pentacyclic skeleton of **1** was constructed in a single step using cooper-catalysed radical domino reaction of cyanamide **53**. The corresponding *N*-benzoylcyanamide **53** was prepared using a two-step sequence from 2-iodo-3-aminomethylquinoline **52** [46] involving the reaction using cyanogen bromide followed by a benzoylation step (Scheme 18). Thus, the treatment of **53** with 10 mol% of heteroleptic copper(I) complex [(DPEphos)(bcp)Cu]PF_6_ in the presence of dicyclohexylisobutylamine as the reductant and K_2_CO_3_ as a base under light irradiation (420 nm) in acetonitrile at room temperature afforded luotonin A (**1**) at a 79% yield. The mechanism of this photoinduced domino transformation involves the generation of radical intermediate **54**, which undergoes 5-exo-dig cyclisation to form the intermediate iminyl radical **55**. This radical **55** then cyclises through a 6-endo-trig process and subsequent aromatisation of in situ-formed **56** provides luotonin A (**1**).

## 3. Summary and Perspective

This review presents the syntheses and biological properties of new derivatives of luotonins A, B, and E. Many new analogues of these natural products, bearing various substituents at different positions on the A, B, C and E rings, have been reported. Some of these derivatives were found to exhibit enhanced topoisomerase I inhibition and anticancer, antifungal, and antiviral activities compared to naturally occurring luotonin A. The broad spectrum of biological properties suggests the potential of these analogues, prompting further systematic study of such molecules and their structural analogues. During the period covered in this review, synthetic strategies based mostly on the Povarov [4+2] cycloaddition and *aza*-Nazarov–Frieländer cyclisation reaction were used for the construction of the 5*H*-pyrrolo[4,3-*b*]pyridine core (B and C rings). The next part of this review discusses advancements in the synthesis of the pentacyclic skeleton of **1** involving the pyrrole ring formation (C ring). Additionally, synthetic methods forming the pyrrolo[1,2-*a*]pyrimidin-4(6*H*)-one fragment (C and D rings) provide access to the desired quinolino[2′,3′:3,4]pyrrolo[2,1-*b*]quinazolin-11(13H)-one scaffold. These approaches typically involve multistep procedures or complex substrates. The main challenge remains to develop an effective strategy for the construction of a pentacyclic backbone by the simultaneous formation of more bonds in a single step. It is anticipated that this could be achieved by exploiting the potential of a Heck-type reaction in new domino sequences, starting from readily/commercially available substrates. Another significant challenge lies in developing suitable methods for industrial applications, e.g., scalable or continuous flow synthesis.

## References

[1] Nomura T., Ma Z.-Z., Hano Y., Chen Y.-J. (1997). Two New Pyrroloquinazolinoquinoline Alkaloids from Peganum nigellastrum. Heterocycles.

[2] Nomura T., Ma Z.-Z., Hano Y., Chen Y.-J. (1999). Two New Quinazoline-Quinoline Alkaloids from Peganum nigellastrum. Heterocycles.

[3] Ma Z.Z., Hano Y., Nomura T., Chen Y.J. (2000). Alkaloids and phenylpropanoids from Peganum nigellastrum. Phytochemistry.

[4] Ma Z., Hano Y., Nomura T., Chen Y. (2004). Novel quinazoline-quinoline alkaloids with cytotoxic and DNA topoisomerase II inhibitory activities. Bioorg. Med. Chem. Lett..

[5] Ma Z., Hano Y., Qiu F., Shao G., Chen Y., Nomura T. (2008). Triterpenoids and Alkaloids from the Roots of Peganum Nigellastrum. Nat. Prod. Commun..

[6] Hano Y., Ma Z., Nomura T. (2005). Luotonin A: A Lead toward Anti-Cancer Agent Development. Heterocycles.

[7] Huang W.P., Liu J.L., Wang C.L. (2009). Progress in the Synthesis of Natural Product Luotonin A and Its Derivatives. Chin. J. Org. Chem..

[8] Michael J.P. (2008). Quinoline, quinazoline and acridone alkaloids. Nat. Prod. Rep..

[9] Cagir A., Jones S.H., Gao R., Eisenhauer B.M., Hecht S.M. (2003). Luotonin A. A naturally occurring human DNA topoisomerase I poison. J. Am. Chem. Soc..

[10] Wall M.E., Wani M.C., Cook C.E., Palmer K.H., McPhail A.T., Sim G.A. (1966). Plant Antitumor Agents. I. The Isolation and Structure of Camptothecin, a Novel Alkaloidal Leukemia and Tumor Inhibitor from Camptotheca acuminata1,2. J. Am. Chem. Soc..

[11] Liang J.L., Cha H.C., Jahng Y. (2011). Recent advances in the studies on luotonins. Molecules.

[12] Haider N., Nuss S. (2012). Weinreb amidation as the cornerstone of an improved synthetic route to A-ring-modified derivatives of luotonin A. Molecules.

[13] Haider N., Meng G., Roger S., Wank S. (2013). An efficient and selective access to 1-substituted and 3-substituted derivatives of luotonin A. Tetrahedron.

[14] Atia M., Bogdán D., Brügger M., Haider N., Mátyus P. (2017). Remarkable regioselectivities in the course of the synthesis of two new Luotonin A derivatives. Tetrahedron.

[15] Zhou H.B., Liu G.S., Yao Z.J. (2007). Short and efficient total synthesis of luotonin A and 22-hydroxyacuminatine using a common cascade strategy. J. Org. Chem..

[16] Dai W., Petersen J.L., Wang K.K. (2006). Synthesis of the parent and substituted tetracyclic ABCD ring cores of camptothecins via 1-(3-aryl-2-propynyl)- 1,6-dihydro-6-oxo-2-pyridinecarbonitriles. Org. Lett..

[17] Nociari M.M., Shalev A., Benias P., Russo C. (1998). A novel one-step, highly sensitive fluorometric assay to evaluate cell-mediated cytotoxicity. J. Immunol. Methods.

[18] Ibric A., Eckerstorfer S., Eder M., Louko I., Tunjic L., Heffeter P., Schueffl H.H., Marian B., Haider N. (2019). Position-Selective Synthesis and Biological Evaluation of Four Isomeric A-Ring Amino Derivatives of the Alkaloid Luotonin A. Molecules.

[19] Ibric A., Battisti V., Deckardt S., Haller A.V., Lee C., Protsch C., Langer T., Heffeter P., Schueffl H.H., Marian B. (2020). A-ring and E-ring modifications of the cytotoxic alkaloid Luotonin A: Synthesis, computational and biological studies. Bioorg. Med. Chem. Lett..

[20] Yang G.Z., Zhang J., Peng J.W., Zhang Z.J., Zhao W.B., Wang R.X., Ma K.Y., Li J.C., Liu Y.Q., Zhao Z.M. (2020). Discovery of luotonin A analogues as potent fungicides and insecticides: Design, synthesis and biological evaluation inspired by natural alkaloid. Eur. J. Med. Chem..

[21] Hao Y., Wang K., Wang Z., Liu Y., Ma D., Wang Q. (2020). Luotonin A and Its Derivatives as Novel Antiviral and Antiphytopathogenic Fungus Agents. J. Agric. Food Chem..

[22] Almansour A.I., Arumugam N., Suresh Kumar R., Mahalingam S.M., Sau S., Bianchini G., Menendez J.C., Altaf M., Ghabbour H.A. (2017). Design, synthesis and antiproliferative activity of decarbonyl luotonin analogues. Eur. J. Med. Chem..

[23] Almansour A.I., Suresh Kumar R., Arumugam N., Bianchini G., Menéndez J.C., Al-thamili D.M., Periyasami G., Altaf M. (2019). Design and synthesis of A- and D ring-modified analogues of luotonin A with reduced planarity. Tetrahedron Lett..

[24] More D.A., Shinde G.H., Shaikh A.C., Muthukrishnan M. (2019). Oxone promoted dehydrogenative Povarov cyclization of N-aryl glycine derivatives: An approach towards quinoline fused lactones and lactams. RSC Adv..

[25] Yadav J.S., Reddy B.V.S. (2002). Microwave-assisted rapid synthesis of the cytotoxic alkaloid luotonin A. Tetrahedron Lett..

[26] Wu F., Dong W., Fan S., Yuan Y., Liang C., Chen A., Yin Z., Zhang Z. (2022). Rapid Synthesis of Luotonin A Derivatives via Synergistic Visible-Light Photoredox and Acid Catalysis. J. Org. Chem..

[27] Kwon S.H., Seo H.A., Cheon C.H. (2016). Total Synthesis of Luotonin A and Rutaecarpine from an Aldimine via the Designed Cyclization. Org. Lett..

[28] Rasapalli S., Sammeta V.R., Murphy Z.F., Huang Y., Boerth J.A., Golen J.A., Savinov S.N. (2019). Synthesis of C-Ring-Substituted Vasicinones and Luotonins via Regioselective Aza-Nazarov Cyclization of Quinazolinonyl Enones. Org. Lett..

[29] Rasapalli S., Sammeta V.R., Murphy Z.F., Golen J.A., Agama K., Pommier Y., Savinov S.N. (2021). Design and synthesis of C-aryl angular luotonins via a one-pot aza-Nazarov-Friedlander sequence and their Topo-I inhibition studies along with C-aryl vasicinones and luotonins. Bioorg. Med. Chem. Lett..

[30] Song Y.C., Wang M.X., Yi Y.Y., Liu Y.T., Zhang W.X., Wang Z.Y., Sun Y.Y., Wu A.X., Zhu Y.P. (2024). FeCl3/KI-Catalyzed Tandem Oxidative Cyclization for Switchable Total Synthesis of Luotonin A, B and Derivatives. Adv. Synth. Catal..

[31] Gonzalez-Ruiz V., Pascua I., Fernandez-Marcelo T., Ribelles P., Bianchini G., Sridharan V., Iniesta P., Ramos M.T., Olives A.I., Martin M.A. (2014). B-ring-aryl substituted luotonin A analogues with a new binding mode to the topoisomerase 1-DNA complex show enhanced cytotoxic activity. PLoS ONE.

[32] Rasapalli S., Huang Y., Sammeta V.R., Alshehry R., Anver F., Shivasankar K., Chavan S.P. (2023). Total and Diverted Total Synthesis of Pyrrolo-Quinazolinone Alkaloids and Their Analogues. ChemistrySelect.

[33] Afanasyev O.I., Podyacheva E., Rudenko A., Tsygankov A.A., Makarova M., Chusov D. (2020). Redox Condensations of o-Nitrobenzaldehydes with Amines under Mild Conditions: Total Synthesis of the Vasicinone Family. J. Org. Chem..

[34] Nagarapu L., Gaikwad H., Bantu R. (2012). TBAHS-Catalyzed Synthesis of 2-Dihydroquinazolin-2-ylquinoline: An Efficient and Practical Synthesis of Naturally Occurring Alkaloids Luotonin A, B, and E. Synlett.

[35] Zhang Y., Zhou Z., Li Z., Hu K., Zha Z., Wang Z. (2022). Iodine-mediated electrochemical C(sp3)-H cyclization: The synthesis of quinazolinone-fused N-heterocycles. Chem. Commun..

[36] Xiang Y., Li H., Wang J., Peng X., Hu C., Luo L. (2021). Design, synthesis, and anticancer activities of 8,9-substituted Luotonin A analogs as novel topoisomerase I inhibitors. Med. Chem. Res..

[37] Rao K.R., Mekala R., Raghunadh A., Meruva S.B., Kumar S.P., Kalita D., Laxminarayana E., Prasad B., Pal M. (2015). A catalyst-free rapid, practical and general synthesis of 2-substituted quinazolin-4(3H)-ones leading to luotonin B and E, bouchardatine and 8-norrutaecarpine. RSC Adv..

[38] Ramamohan M., Raghavendrarao K., Sridhar R., Nagaraju G., Chandrasekhar K.B., Jayapraksh S. (2016). A concise approach to substituted Quinazolin-4(3H)-one natural products catalyzed by Iron(III) Chloride. Tetrahedron Lett..

[39] Wang C., Qian P.-C., Chen F., Cheng J. (2020). Rhodium-catalyzed [4+1] annulation of sulfoxonium ylides: Sequential ortho-C H functionalization/carbonyl α-amination toward polycyclic quinazolinones. Tetrahedron Lett..

[40] Wang R.X., Du S.S., Wang J.R., Chu Q.R., Tang C., Zhang Z.J., Yang C.J., He Y.H., Li H.X., Wu T.L. (2021). Design, Synthesis, and Antifungal Evaluation of Luotonin A Derivatives against Phytopathogenic Fungi. J. Agric. Food Chem..

[41] Mondal S., Sultana F., Dutta S., Mondal M.A. (2023). Synthesis of Luotonin and Rutaecarpine Analogues by One-Pot Intramolecular Dehydrogenative Cross-Coupling and Benzylic C−H Oxidation, and In Vitro Cytotoxicity Assay. ChemistrySelect.

[42] Kagawa N., Toyota M., Nishimura K., Abe S. (2018). Concise Approach to Mono- and Disubstituted Luotonin A Analogs and Their Cytotoxicity Test. Heterocycles.

[43] Waring A., Toyota M., Komori C., Ihara M. (2003). An efficient total synthesis of the pyrroquinazolinoquinoline alkaloid, Luotonin A, employing an intramolecular hetero Diels–Alder reaction. Arkivoc.

[44] Toyota M., Natsuki K., Shindo T. (2012). Practical Total Synthesis of Luotonin A by Intramolecular Double Hetero Diels-Alder Reaction. Heterocycles.

[45] Evano G., Baguia H., Deldaele C., Romero E., Michelet B. (2018). Copper-Catalyzed Photoinduced Radical Domino Cyclization of Ynamides and Cyanamides: A Unified Entry to Rosettacin, Luotonin A, and Deoxyvasicinone. Synthesis.

[46] Servais A., Azzouz M., Lopes D., Courillon C., Malacria M. (2007). Radical cyclization of N-acylcyanamides: Total synthesis of luotonin A. Angew. Chem. Int. Ed..

